# Trends in the incidence of cancers related to HIV-AIDS in Harare, Zimbabwe 1990-2019

**DOI:** 10.1093/jnci/djaf194

**Published:** 2025-07-16

**Authors:** Eric Chokunonga, Rudo Makunike-Mutasa, Margaret Borok, Mike Z Chirenje, Ntokozo Ndlovu, Justice Mudavanhu, Apollo Tsitsi, Biying Liu, Donald Maxwell Parkin

**Affiliations:** Zimbabwe National Cancer Registry, Parirenyatwa Group of Hospitals, Harare, Zimbabwe; Zimbabwe National Cancer Registry, Parirenyatwa Group of Hospitals, Harare, Zimbabwe; Department of Laboratory Diagnostic and Investigative Sciences, Faculty of Medicine and Health Sciences, University of Zimbabwe, Harare, Zimbabwe; Zimbabwe National Cancer Registry, Parirenyatwa Group of Hospitals, Harare, Zimbabwe; Department of Internal Medicine, Faculty of Medicine and Health Sciences, University of Zimbabwe, Harare, Zimbabwe; Zimbabwe National Cancer Registry, Parirenyatwa Group of Hospitals, Harare, Zimbabwe; Department of Obstetrics, Gynaecology and Reproductive Science, University of California San Francisco, San Francisco, CA, United States; Zimbabwe National Cancer Registry, Parirenyatwa Group of Hospitals, Harare, Zimbabwe; Department of Oncology, Medical Physics and Imaging Sciences, Faculty of Medicine and Health Sciences, University of Zimbabwe, Harare, Zimbabwe; Department of NCDs, Ministry of Health and Child Care, Harare, Zimbabwe; Ministry of Health and Child Care, HIV Program, Harare, Zimbabwe; African Cancer Registry Network, Oxford, United Kingdom; International Agency for Research on Cancer, Lyon, France; Nuffield Department of Population Health, University of Oxford, Oxford, United Kingdom

## Abstract

**Background:**

HIV prevalence in Harare reached a maximum of around 33% of adults in 1995, before falling to 12% in 2019. We examine trends in the incidence of Kaposi sarcoma (KS), non-Hodgkin lymphoma (NHL), Hodgkin lymphoma, and squamous cell conjunctival cancers (SCCCs) in the population of Harare in relation to changes in HIV prevalence, and the increasing availability and use of antiretroviral therapy (ART).

**Methods:**

Data from the population-based cancer registry of Harare are used to calculate incidence rates for the Black (African) population for the years 1990-2019.

**Results:**

Incidence of KS increased to a peak in the late 1990s, after which rates declined, especially at younger ages. Mean age at diagnosis increased by about 8 years in men and 6 years in women. SCCC shows a similar trend to that of KS, with a dramatic 10-fold increase in incidence, followed by an equivalent fall. Although Hodgkin lymphoma showed no change in incidence over the 30-year period, rates of NHL progressively increased. Incidence in younger adults (aged younger than 44) stabilized after about 2001 but continued to increase in older individuals.

**Conclusions:**

The availability of high-quality cancer registry data over a long period has provided a unique opportunity to study the effects of the epidemic of HIV-AIDs and of ART availability on the risk of cancer in an African population. As HIV prevalence fell and ART coverage expanded, incidence of KS and SCCC declined, whereas for NHL the trends suggest that long-term infection with HIV may pose an increased risk, despite ART.

## Introduction

In its 2012 evaluation of the association between HIV infection and cancer, the International Agency for Research on Cancer (IARC)^1^ concluded, “There is sufficient evidence in humans for the carcinogenicity of infection with HIV-1. Infection with HIV-1 causes cancer of the cervix, anus, and conjunctiva, and Kaposi sarcoma, non-Hodgkin lymphoma, and Hodgkin lymphoma.” It also noted that a positive association had been observed between infection with HIV-1 and cancer of the vulva and vagina, penis, hepatocellular carcinoma, and non-melanoma skin cancer. At the time, almost all the evidence, from follow-up of cohorts of people living with HIV (PLHIV), was from Europe, North America, and Australia. Since then, results have been reported from cohorts in Africa: in Uganda,^2^ South Africa^3^ and Rwanda,^4^ as well as a small study in Nigeria.^5^ They confirm elevated risks in PLHIV for Kaposi sarcoma, non-Hodgkin lymphoma, and cancers of the cervix (so-called AIDS-defining cancers), as well as Hodgkin lymphoma and squamous cell carcinoma of conjunctiva (SCCC) and, less regularly, cancers of the vulva, penis, and anus.

Almost all of the cancers related to AIDS are caused by infection with an oncogenic virus; HIV-1 increases the cancer risk indirectly, primarily by immunosuppression. Thus, the risk of developing cancer is closely related to markers of immunosuppression, such as the CD4 count.^6^ Accordingly, treatment with antiretrovirals, which diminishes immunosuppression and increases CD4 counts, might be expected to reduce the risk of these cancers. A decrease in the incidence rates of certain HIV-related cancers, such as Kaposi sarcoma and non-Hodgkin lymphoma, has indeed been observed as antiretroviral therapy (ART) became more widely available.^7^^,^^8^ Similarly, Metekoua et al.^9^ found a decreasing trend in SCCC incidence rates between 2004 and 2014 among PLHIV in South Africa.

Zimbabwe is one of the countries in Africa that have been severely affected by the HIV epidemic, with the prevalence of infection increasing to a maximum of around 23% among adults (15-49) in 1997, before falling to 18.4% in 2005, and 12.8% in 2019.^10^ These changes in HIV prevalence, as well as the increasing availability and use of ART, may be reflected in the trends of AIDS-related cancers. The Zimbabwe National Cancer Registry (ZNCR) began operations in Harare in 1986, with acceptably complete coverage of the population of the city of Harare achieved in 1990.^11^ As a result, it is 1 of only 2 cancer registries in Africa able to document the evolution of cancer patterns over a substantial period of time (the other being the Kampala cancer registry in Uganda).^12^ In previous articles we have examined trends in cancers related to AIDS in the Black (African) population of Harare for the period 1990-1995^13^ and for a 20-year time period (1991-2010).^14^ In the current study we examine time trends for the 30-year period 1990-2019, focusing on some of the major cancers shown to be related to infection with HIV-AIDS in Africa: Kaposi sarcoma, non-Hodgkin lymphoma, Hodgkin lymphoma, and conjunctival cancers. Trends in the incidence of female genital cancer (cervix, vulva, and vagina) are the subject of a separate analysis.

## Methods

The methods employed by the ZNCR have been described previously.^15^^,^^16^ Briefly, the registry is situated in a major referral hospital for the northern part of the country (Parirenyatwa Group of Hospitals). It collects information on cancer patients diagnosed and treated in all hospitals and clinics in the city of Harare, as well as pathology laboratories, both by voluntary notification from certain institutions and by staff visits. Cancer notification forms are filled in for each patient. Information collected includes patient demographic data, as well as details of the tumor, the basis of diagnosis, its treatment, the source(s) of information on each case, and follow up (date of last contact or death). All cases are registered, including not only those with morphological verification (histology or cytology) of the diagnosis but also those diagnosed clinically, at surgery, by medical imaging, or by specific biochemical or immunological tests. Information on the abstract forms is coded and entered into the computer using the CanReg5 cancer registration software provided by the IARC. Tumor site and morphology are coded according to the third edition of the International Classification of Diseases for Oncology (ICD-O-3).^17^ For tabulation of results, these were converted to the 10th revision of the ICD (ICD-10).

Medical certification of death (by cause) is relatively complete for the city of Harare, and the registry uses death registrations as an important source of information on cases that may have been missed by the registration process. Deaths with cancer (ICD-10 C00-C96) as an underlying or contributory cause of death are matched with the registry database, and if not already registered, the case is followed up to obtain additional information on the diagnosis and management of the cancer. If this proves fruitless, the case is registered on the basis of the death certificate only (DCO).

### Population

Population censuses were performed in 1992, 2002, 2012, and 2022; for these years, the population of Harare was available by sex, ethnic group, and 5-year age group from ZimStat (the Zimbabwe Statistical Agency). Annual intercensal estimates were prepared, assuming a constant rate of growth within age groups between census counts. Population pyramids for the Black population at the beginning (1992 census) and end (2022 census) of the period studied are shown as a Figure S1.

### Statistical methods

Incidence rates were calculated for the Black population by 5-year age groups and sex, for each year (1990-2019), and for six 5-year time periods: 1990-1994, 1995-1999, 2000-2004, 2005-2009, 2010-2014, 2015-2019. Age standardized rates (ASRs) were calculated using the World Standard population.^18^ Rates are expressed as per 10^5^ (per 100 000 person years).

Temporal trends over the whole 30-year period were examined by fitting a regression line to the log-transformed age-standardized incidence rates. From this, we calculated the average annual percentage change (AAPC) as the slope of the regression line, together with its 95% confidence interval.^19^

Two widely used indicators of data quality^20^—the percentage of cases with morphological verification (histology or cytology) of diagnosis (MV%) and the percentage of cases registered with information from a death certificate onlyDCO (DCO%) were calculated for each sex and the same periods.

Graphs of time trends in rates use 3-year moving average values of rates.

## Results

Table 1 shows the total number of cases registered, the ASRs in each of the 5-year periods, and the AAPC in incidence over the period 1990-2019 for Kaposi sarcoma, SCCC, Hodgkin lymphoma, and non-Hodgkin lymphoma, in males and females.

**Table 1.** Total number of cases registered, age standardized incidence rates in each of the 5-year periods, and the average annual percentage change (AAPC) in incidence over the period 1990-2019.

			ASRs (per 10^5^)							
Males	ICD 10	Total cases	1990-1994	1995-1999	2000-2004	2005-2009	2010-2014	2015-2019	1990-2019	AAPC (95% CI) 1990-2019
Kaposi sarcoma	C46	5950	34.4	64.7	55.2	27.3	20.9	13.3	34.6	–^a^
Squamous cell carcinoma of conjunctiva		396	0.2	2.7	5.2	1.6	3.5	1.4	2.4	2.17 (−2.69 to 7.03)
Hodgkin lymphoma	C81	142	0.82	0.66	1.19	0.60	0.86	0.80	0.82	−0.27 (−2.55 to 2.00)
Non-Hodgkin lymphoma	C82-85	1382	4.2	7.8	9.8	8.7	14.6	13.1	10.1	3.95 (2.64 to 5.26)
Females										
Kaposi sarcoma	C46	2919	11.8	28.8	31.2	15.5	11.8	5.7	16.4	–^a^
Squamous cell carcinoma of conjunctiva		412	0.7	3.2	4.5	2.5	3.1	2.0	2.8	0.77 (−1.68 to 3.22)
Hodgkin lymphoma	C81	95	0.34	0.62	0.77	0.51	0.73	0.65	0.64	−0.89 (−3.98 to 2.19)
Non-Hodgkin lymphoma	C82-85	1146	4.2	7.7	8.0	9.4	11.4	10.3	9.2	3.38 (2.13 to 4.62)

a 30-Year AAPC was not calculated because of the non-monotonic trend as shown in Figure 1.

Over the 30-year period, there has been an overall decline in the incidence of Kaposi sarcoma, both in males and females, but this overall trend hides the fluctuation in incidence, with a smooth curve of increase to a peak, followed by a decrease to around 2006. In males, the peak incidence is in 1998-1999 (ASR of 70.5 per 10^5^). In females the peak occurs a little later (1999-2001), with a maximum incidence was about half that in males (35.3 per 10^5^) (Figure 1). In the past 10 years (2010-2019) there is a simple linear downward trend [AAPC in males = −9.49% (95% confidence interval [CI] = −13.01 to −5.97), in females = −11.64% (95% CI = −14.93 to −8.34)].

**Figure 1.**
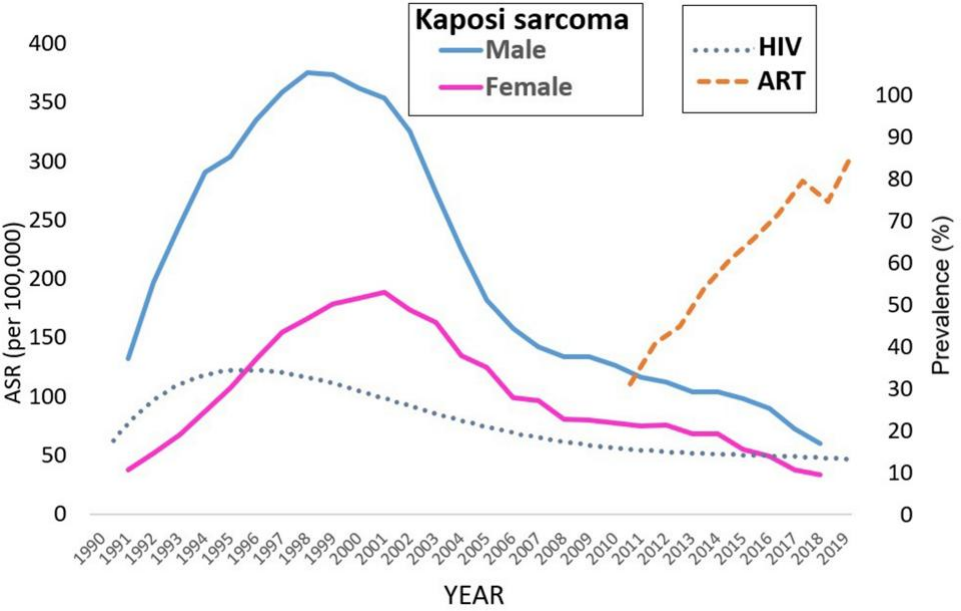
Age-standardized incidence rates (per 10^5^) for Kaposi sarcoma in males and females 1990-2019. Values (Left) shown are 3-year moving average rates. Right: Adult HIV prevalence (%) in Harare (1990-2019) and percentage of people living with HIV receiving antiretroviral therapy (ART) in Zimbabwe (2010-2019).^10^

Figure 1 also shows the prevalence of HIV infection in adults (15-49) in Harare over the same time period, as well as coverage by ART (as the percentage of PLHIV receiving ART) in Zimbabwe since 2010.^10^

**Figure 2.**
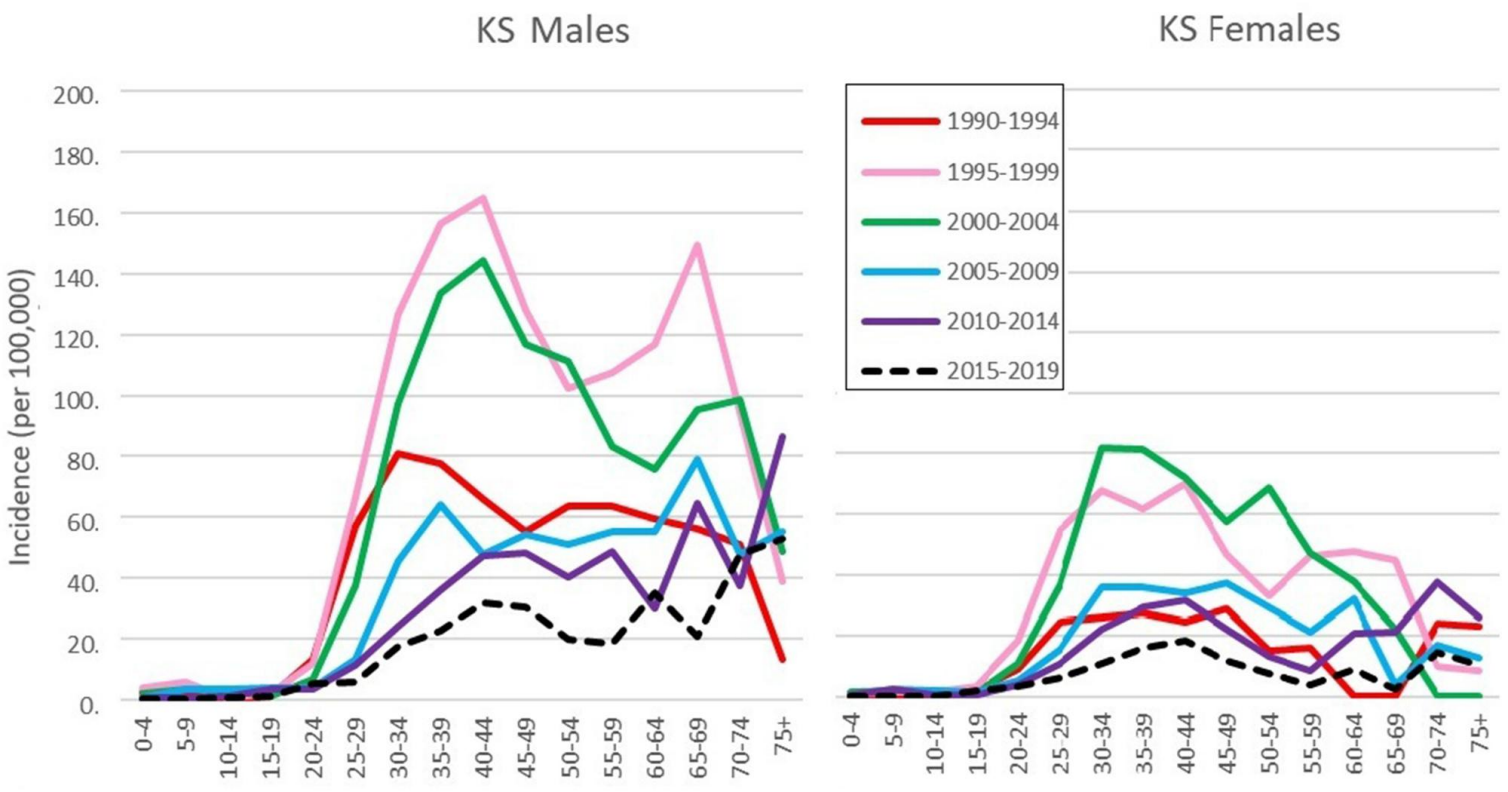
Age-specific incidence rates (per 10^5^) of Kaposi sarcoma (KS), according to period of diagnosis, for males (3a) and females (3b).

Looking at age-specific incidence over time (Figure 2) shows that the changes in age-standardized rates are the result of the emergence of a peak in rates in young adults, around 30-49 in men, rather younger in women (25-44), most marked in the period 1995-2001 (a little later in women than in men). It is noteworthy that there is also a second peak in incidence for males, in the age range 60-69, which is also enhanced in the time period 1995-2004. In the most recent decade (2000-2019), the highest rates of Kaposi sarcoma are in the oldest age groups. As a result of these trends, the mean age at diagnosis increases over time in both men (36.3 in 1990-1994 to 42.0 in 2014-2019) and in women (31.4 in 1990-1994 to 37.1 in 2014-2019).

**Figure 3.**
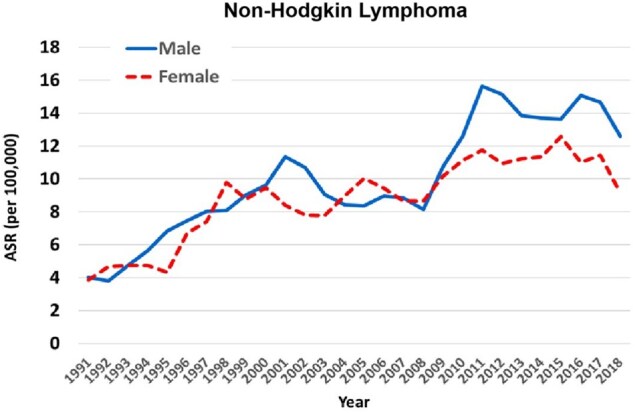
Trends in age standardized rates (ASRs) (per 10^5^) of non-Hodgkin lymphomas (ICD 10 C82-85 and C96) in males and females (3-year moving averages 1991-2018).

The incidence of non-Hodgkin lymphoma (ICD-10 C82-85, C96) has increased over the whole period in both sexes: AAPC is 3.9 (95% CI = 2.6 to 5.3) in males and 3.4 (95% CI = 2.1 to 4.6) in females (Table 1), and this rate of increase appears to be relatively constant (Figure 3).

**Figure 4.**
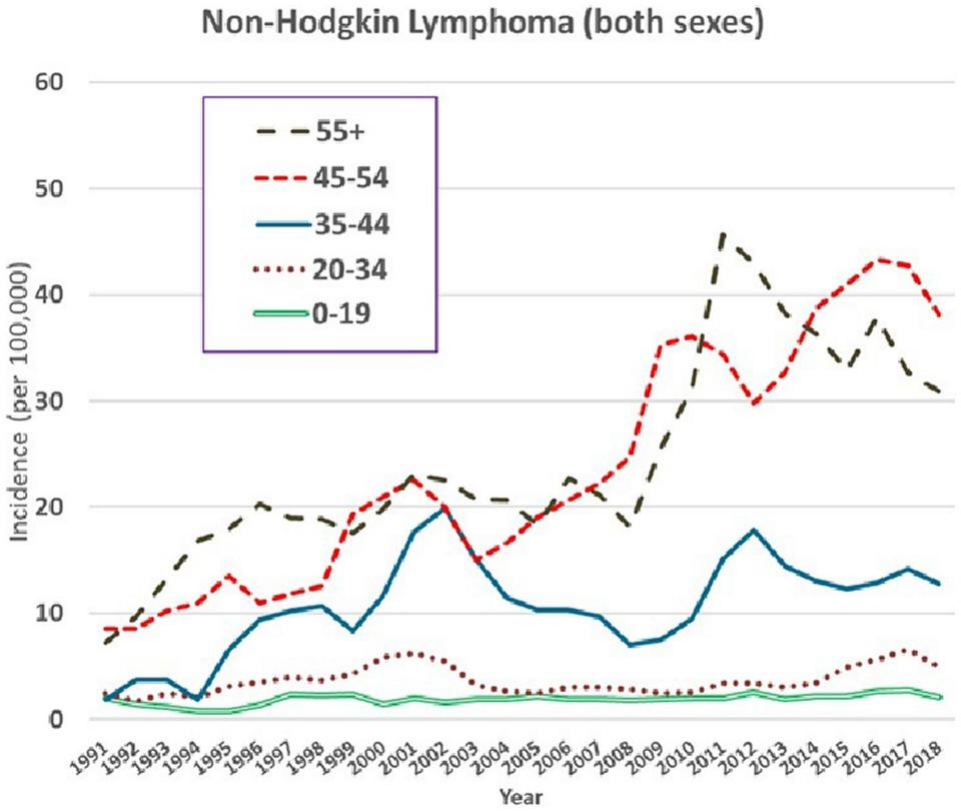
Trends in age-specific incidence rates (per 10^5^) of non-Hodgkin lymphomas (ICD 10 C82-85 and C96) in both sexes (3-year moving averages 1991-2018).

Looking at the trends in age-specific rates (Figure 4), there is an increase in incidence in younger age groups between 1990 and 2001/2, after which they are relatively stable, but in older age groups (45+), the rates continue to increase.

**Figure 5.**
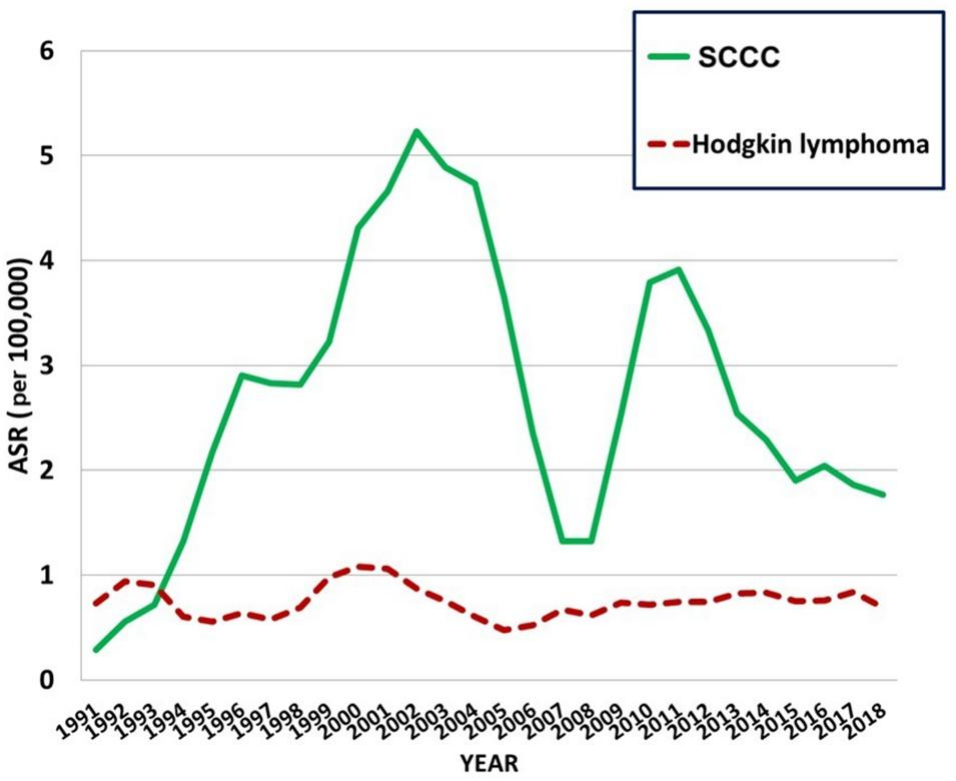
Trends in age standardized rates (per 10^5^) of squamous cell carcinomas of conjunctive (SCCCs) and Hodgkin lymphomas in both sexes (3-year moving averages 1991-2018).

Figure 5 shows the trends in age-standardized incidence rates of SCCC and of Hodgkin lymphoma (both sexes combined). For SCCC, incidence rates rise to a maximum in 2002, and then decline, although there is an enormous dip in the rates between 2005 and 2010. For Hodgkin lymphoma, ASRs increase with age, without the young adult peak noted in high-income populations. There has been no statistically significant change in incidence over the past 30 years, confirmed by the nonsignificant AAPCs in Table 1.

**Figure 6.**
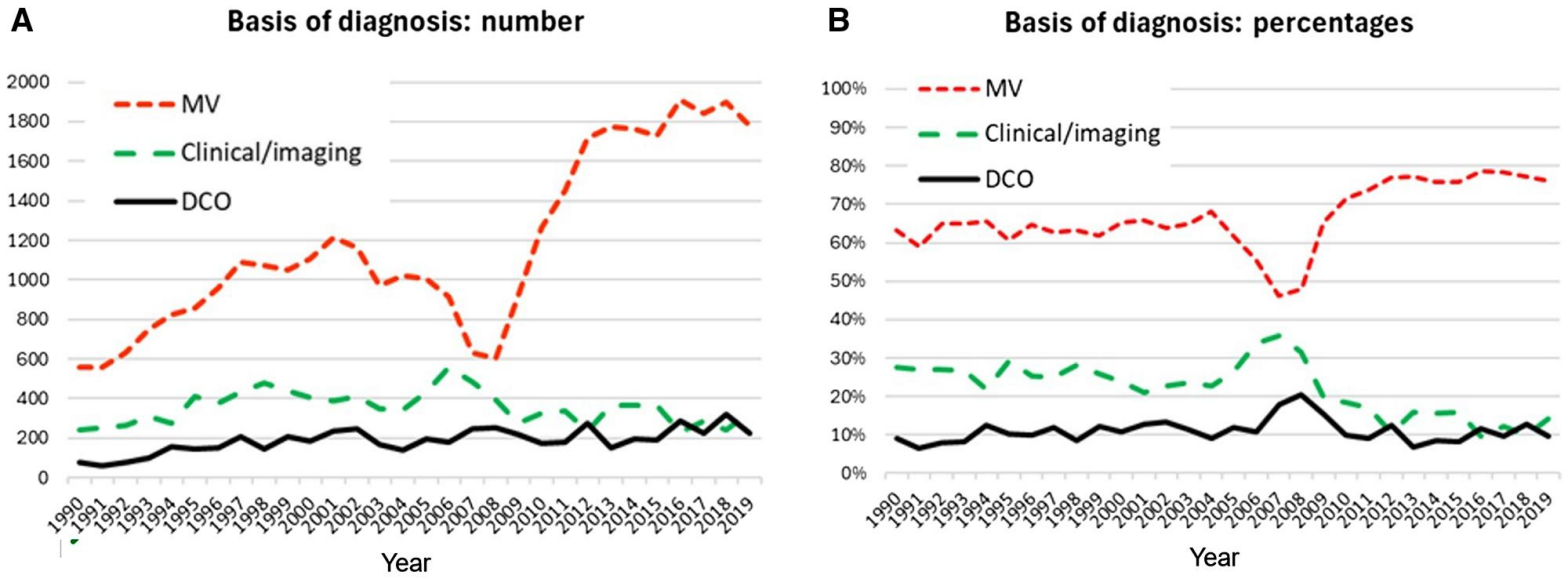
Most valid basis of diagnosis of registered cases 1990-2019 as (**A**) numbers of registrations and (**B**) percentage of registrations. Abbreviations: Clinical/Imaging: = clinical examination (endoscopy, surgery, etc) and imaging; DCO = death certificate only; MV: morphologically verified (histology or cytology).

Figure 6 shows temporal trends in 2 indicators of data quality^20^—the number (and percentage) of cases diagnosed with a morphological verification of the diagnosis (MV), and cases registered on the basis of information on a DCO. The MV% and DCO% remain relatively constant until about 2005. In 2006-2008 the number of cases registered and the percentage of cases morphologically verified show a decrease, before the MV% and DCO% stabilize after 2009.

## Discussion

In the evaluation of time series data, it is essential to avoid artifacts resulting from variation in the degree of completeness of registration of incident cancer cases in the period under consideration. Results from the registry have been published in six successive volumes of Cancer Incidence in Five Continents (CI5) from volumes VII (1990-1992) to volume XII (2013-2017)^21^; these volumes present “high-quality statistics on cancer incidence from population-based cancer registries around the world.” The decrease in cases diagnosed (and registered), and the lower percentage of those with morphological verification of diagnosis in 2006-2008 (Figure 6) is a consequence of a very difficult time—politically, economically, and socially—for Zimbabwe. The severe economic challenges experienced in the country adversely affected the health-care delivery system, which impacted on diagnosis and treatment of cancers cases.

As a result, the calculated incidence rates show that registration was incomplete during this period (notably for the 3 years 2007-2009). Although this will appear as a dip in the curve of incidence over time, because it is toward the middle of the 30-year period, it does not much affect the overall trends shown in Table 1. We tested this by recalculating the AAPC, omitting the 3 years 2007-2009. Although the actual value of the AAPC was changed slightly, in no case was the statistical significance of the trend (from the null value) different (Table S1), so we have retained the values incorporating the 3 years concerned.

In addition to completeness of ascertainment, a valid estimation of incidence rates requires that accurate population denominators are available. For Harare, census counts (by age group, sex, and race) of the population were available for 1992, 2002, 2012, and 2022, so annual estimates (interpolations) should be reasonably accurate. Updating population-at-risk with more recent (census) data means that there will often be discrepancies between the incidence rates shown in this article and those published in past volumes of CI5, or our previous publication on 20-year trends.^14^

In the 30 years after 1990, there were quite dramatic changes in incidence affecting those cancers related to infection with HIV, particularly Kaposi sarcoma, SCCC, and non-Hodgkin lymphoma. HIV prevalence in Zimbabwe increased throughout the last years of the 1980s to reach a maximum of around 33% in adults (aged 15-49) in Harare in 1995, before falling to 15% in 2009 and 12% in 2019 (Figure 1).^10^ Incidence rates of infection in Zimbabwe are also falling (from 6.4% per 1000 uninfected population in 2010 to 2.8% in 2019). Incidence is highest in the young; maximum rates are in age groups 20-24 in women at ages 25-29 in men.^10^

Mortality from HIV-AIDS has been dramatically reduced by the availability of ART. The ART program was launched by the Ministry of Health and Child Welfare in 2004, and by 2019, it was estimated that 84.7% of all people living with HIV were receiving antiretroviral therapy (Figure 1).^10^^,^^22^

As a result, the age-specific prevalence of persons living with HIV has shifted; the peak was at ages 25-34 in 2000-2004^23^ and by 2020 the maximum prevalence was at ages 45-49 in women (33.3%) and at ages 50-54 (at 30.9%) in men.

These changes have been reflected in cancers associated with HIV-AIDS.

Kaposi sarcoma, one of the major AIDS defining malignancies, is caused by the Kaposi Sarcoma–associated Herpes Virus, with a synergism between this virus and HIV. In Africa, Kaposi sarcoma represents one of the most common cancers in HIV-infected individuals. Before the epidemic of HIV-AIDS, Kaposi sarcoma in Africa was of the typical “endemic” pattern, involving the skin, particularly the legs, and affecting principally male individuals, with the risk rising progressively with age.^15^ There has been an enormous increase in incidence of Kaposi sarcoma in Harare since the first report on cancer in Harare (for the years 1986-1989^24^). These changes are the result of the evolution of the epidemic of HIV-AIDS in Zimbabwe, and the age-specific incidence of Kaposi sarcoma was noted to correspond closely to the age-specific reporting rates for AIDS.^13^

Incidence rates of Kaposi sarcoma have followed a similar trajectory to the decline in prevalence of HIV, but with the curve advanced by some 3-4 years. This decline in incidence, resulting from either the natural dynamics of HIV epidemics or the impact of interventions, is most marked in the younger age-groups where most individuals have had little previous exposure to infection and HIV prevalence reflects recent incidence. In older age-groups, aging of persons infected at younger ages tends to offset the effects of mortality and reduce HIV incidence.^25^ The fall in incidence of Kaposi sarcoma in men since 1998 and in women since 2000 has similarly been more marked in the younger age groups, resulting in an increase in the mean age at onset. The fall has almost certainly been accelerated by the increasing availability of ART, which suppresses manifestations of AIDS (particularly Kaposi sarcoma) in HIV-positive subjects.^26-28^

SCCC is relatively common in populations in sub-Saharan Africa.^29^ The observation of relatively high incidence rates in tropical Africa was made 50 years ago^30^ and led to the investigation of possible etiological factors. With the high occurrence in equatorial regions, an association with exposure to UV irradiation was suspected and has been confirmed at ecological^31^^,^^32^ and (less certainly) individual level.^33^ More striking, however, is the clear association with infection with HIV-AIDS. The evidence has been reviewed by IARC, who note the consistent marked increase in risk in persons infected with HIV-1, with a relative risk of about 10.^1^ The onset of the epidemic of HIV-AIDS was accompanied by a marked increase in incidence of this cancer.^13^^,^^34^ The incidence in the Harare population increased 10-fold between 1991 and 2002, but problems of availability of diagnosis and therapy almost certainly underlie the dramatic dip in registrations between 2005 and 2010; without this, the pattern is one of a slow decline in incidence to the level observed at the beginning of the period in the early 1990s. There seems to be little information on the effects of ART therapy on the risk of SCCC in cohorts of PLHIV (probably because it is a much less common manifestation of HIV infection in non-African populations^35^), but incidence rates in a South African cohort of PLHIV were lower in subjects with lower CD4 counts.^9^

The incidence of non-Hodgkin lymphomas has also increased during the 30-year period studied, although among younger adults, aged younger than 45, there has been little change in the incidence rate since 2001/2002. This may also be associated with a declining prevalence of HIV infection in this age group. The risk of non-Hodgkin lymphoma in PLHIV is related to the level of immunosuppression, as reflected by CD4 count^6^^,^^36^ and there has been a decrease in the incidence of systemic and central nervous system non-Hodgkin lymphoma among PLHIV following the introduction of ART.^28^^,^^37^ The risk of non-Hodgkin lymphoma in PLHIV is reduced by treatment with ART (although to a much lesser degree than the risk of Kaposi sarcoma).^38^ The pattern of increasing incidence of non-Hodgkin lymphoma in older age groups is compatible with the risk of non-Hodgkin lymphoma being related to the duration of infection with HIV, and the increasing numbers of persons in these age groups with long-standing HIV infection (as reflected in the increasing prevalence).

The incidence of Hodgkin lymphoma in Harare is low by global standards, but very close to the mean for sub–Saharan Africa.^39^ Although the risk of has been shown to be increased by HIV infection, no trend in incidence over the period can be discerned in Harare. In this respect, long-term time trends in the USA do not show any changes that might be attributed to the prevalence of HIV infection (or treatment with ART).^40^ The effect of ART on risk of Hodgkin lymphoma in PLHIV is also unclear.^41^

## Conclusions

The epidemic of HIV-AIDS in Zimbabwe reached its peak in 1997; since then the incidence of new infections has been declining, and the prevalence of PLHIV has decreased. With the increasing availability of ART, and consequent dramatic improvement in survival, the cohort of PLHIV has been getting progressively older. These changes, and the change in risk of cancer due to improved immune function in PLHIV receiving ART, are reflected in the age-specific incidence of cancers related to HIV. Sub-Saharan Africa is the region of the world most affected by the HIV epidemic, as well as the least well-endowed with disease surveillance systems. The availability of a high-quality population-based cancer registry in Zimbabwe has permitted the careful study of effects of the changes in HIV prevalence and availability of treatment on the risks of cancer in the population. This is a tribute to all those involved in setting up, funding, running, and supporting this cancer registry over the almost 40 years of its existence and, hopefully, an encouragement to health services planners and administrators elsewhere on the continent.

## Supplementary Material

djaf194_Supplementary_Data

## Data availalility

The data that support the findings of our study are available on request to the Zimbabwe National Cancer Registry. All data requests will be evaluated by the ZNCR advisory committee.
