# Supplementary material for: Trends in the incidence of cancers related to HIV-AIDS in Harare, Zimbabwe 1990-2019
Source: J Natl Cancer Inst. 2025 Jul 16;117(10):2096–102. doi: 10.1093/jnci/djaf194 (PMC12505126; doi:10.1093/jnci/djaf194)

**Supplementary Material**

**TABLE S1**

Average annual percentage change (AAPC) in age standardised incidence using all data (1990-2019) and omitting years 2007-2009

| **MALES** | **ICD 10** | **AAPC (95% c.i.)**  **1990-2019** | **AAPC (95% c.i.)**  **Omitting 2007-2009** |
| --- | --- | --- | --- |
| **SCCC** | - | 2.17 (-2.69, 7.03) | 3.67 (-1.30, 8.65) |
| **Hodgkin lymphoma** | C81 | -0.27 (-2.55, 2.00) | -0.39 (-3.32, 2.43) |
| **Non-Hodgkin lymphoma** | C82-85 | 3.95 (2.64, 5.26) | 4.74 (3.21, 6.27) |
| **FEMALES** |  |  |  |
| **SCCC** | - | 0.77 (-1.68, 3.22) | -1.33 (-1.48, 4.14) |
| **Hodgkin lymphoma** | C81 | -0.89 (-3.98, 2.19) | -1.27 (-5.04, 2.50) |
| **Non-Hodgkin lymphoma** | C82-85 | 3.38 (2.13, 4.62) | 4.00 (2.50, 5.49) |

**FIGURE S1**

Population Pyramids for the black (African) population of Harare (1992 and 2022 census).


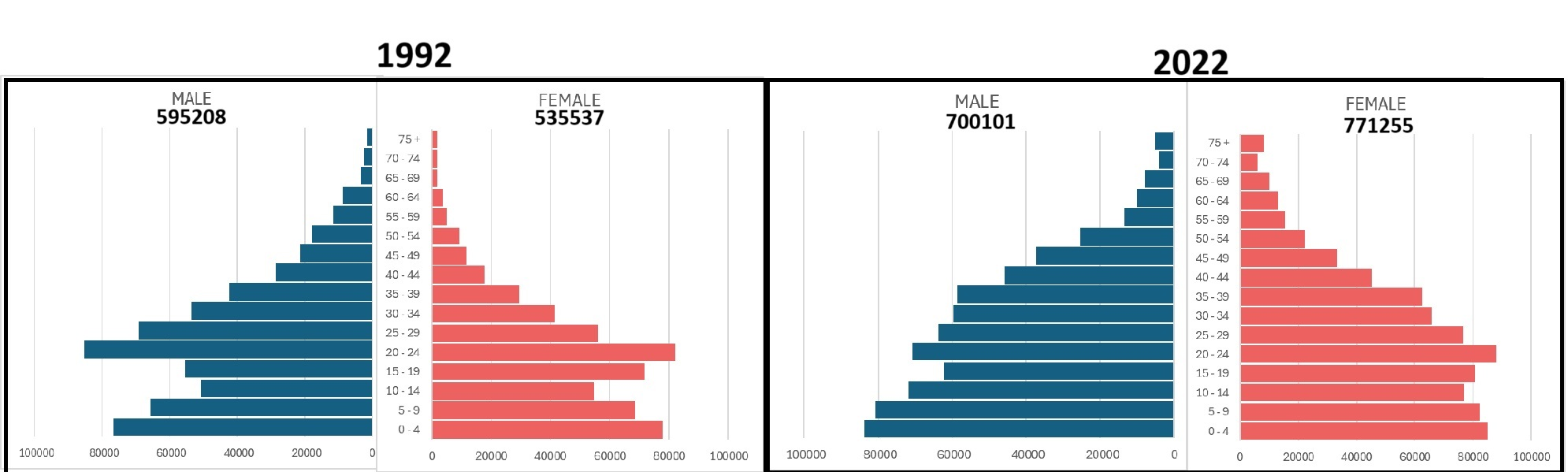

Supplement: djaf194_Supplementary_Data [file djaf194_supplementary_data.zip › Supplementary Material r3.docx]
